# Herbal/Natural Compounds Resist Hallmarks of Brain Aging: From Molecular Mechanisms to Therapeutic Strategies

**DOI:** 10.3390/antiox12040920

**Published:** 2023-04-13

**Authors:** Juhui Qiao, Chenxi Wang, Yu Chen, Shuang Yu, Ying Liu, Shiting Yu, Leilei Jiang, Chenrong Jin, Xinran Wang, Peiguang Zhang, Daqing Zhao, Jiawen Wang, Meichen Liu

**Affiliations:** 1 Northeast Asia Research Institute of Traditional Chinese Medicine, Changchun University of Chinese Medicine, Changchun 130117, China; 2 School of Pharmacy, Changchun University of Chinese Medicine, Changchun 130117, China; 3 Changchun Institute of Optics, Fine Mechanics and Physics, Chinese Academy of Sciences, Changchun 130033, China; 4 Division of Cardiovascular Medicine, Department of Medicine, Solna, Karolinska Institutet, 171 76 Stockholm, Sweden

**Keywords:** age-related diseases, herbal, natural compounds, brain, oxidative modifier

## Abstract

Aging is a complex process of impaired physiological integrity and function, and is associated with increased risk of cardiovascular disease, diabetes, neurodegeneration, and cancer. The cellular environment of the aging brain exhibits perturbed bioenergetics, impaired adaptive neuroplasticity and flexibility, abnormal neuronal network activity, dysregulated neuronal Ca^2+^ homeostasis, accumulation of oxidatively modified molecules and organelles, and clear signs of inflammation. These changes make the aging brain susceptible to age-related diseases, such as Alzheimer’s and Parkinson’s diseases. In recent years, unprecedented advances have been made in the study of aging, especially the effects of herbal/natural compounds on evolutionarily conserved genetic pathways and biological processes. Here, we provide a comprehensive review of the aging process and age-related diseases, and we discuss the molecular mechanisms underlying the therapeutic properties of herbal/natural compounds against the hallmarks of brain aging.

## 1. Introduction

The human aging process is extremely complex and is characterized by a time-dependent decline in physiological functions. Although advances in medicine have increased the average lifespan, an increase in “healthy lifespan” has not yet been achieved, and age-related diseases are major socioeconomic and health care burdens. When an organism ages, all biological systems, from cells to tissues to organs, undergo changes, especially the brain [1]. According to traditional Chinese medicine theory, “the brain is the sea of medulla and the house of the Elemental God”, that is, the brain is composed of the medulla and dominates the life activities of the human body. Modern scientific studies have shown that impaired myelin structure, atrophy of neurons, massive intracellular deposition of lipofuscin, reduced synaptic connections and neurotransmitters, and impaired ability to receive and transmit information are major features of age-related neurodegenerative diseases [2]. Notably, these nervous system changes are precursors of the age-related decline in all other physiological systems and play a major role in the progression of these systemic impairments.

Aging is the major risk factor for late-onset Alzheimer’s disease (AD), which accounts for more than 95% of cases [3]. Parkinson’s disease (PD) is thought to be caused by specific factors (e.g., genetic mutations, hereditary factors), and is characterized by the abnormal aggregation of alpha-synuclein (α-Syn) and the degeneration of nigrostriatal dopaminergic neurons, which are strongly associated with aging [4]. Due to the aging of the central nervous system and the intractability of neurodegenerative lesions, there is still a lack of effective drugs to slow disease progression. Therefore, exploring the mechanisms of brain aging and developing drugs that effectively delay brain aging and protect brain tissues are of great scientific and socioeconomic importance for reducing the incidence of neurodegenerative diseases, improving the quality of life of the elderly, and for achieving healthy aging.

In recent years, Chinese traditional herbs/natural compounds have become a research hotspot in aging research. Ginseng (or ginseng active ingredients) has been characterized to delay age-related diseases such as enhanced cognitive performance, improved cardiovascular and immune function, and extended healthy lifespan in multimodal organisms [5,6,7]. In the brain, curcumin (the yellow polyphenolic compound extracted from *Curcuma longa*) is known for its anti-protein aggregation and neuroprotective activities, as well as its anti-inflammatory, anti-tumor, and immunomodulatory properties [8,9]. Although there is a growing body of evidence that herbal/natural compounds protect against brain aging and disease, the mechanisms underlying their neuroprotective actions are largely unknown.

In this review article, we summarize the role of herbal/natural compounds in the treatment of brain aging-induced diseases, based on the molecular characteristics of the aging brain, in the hope that it may help advance research into age-related diseases.

## 2. Structural and Functional Characteristics of Brain Aging

**Figure 1:**
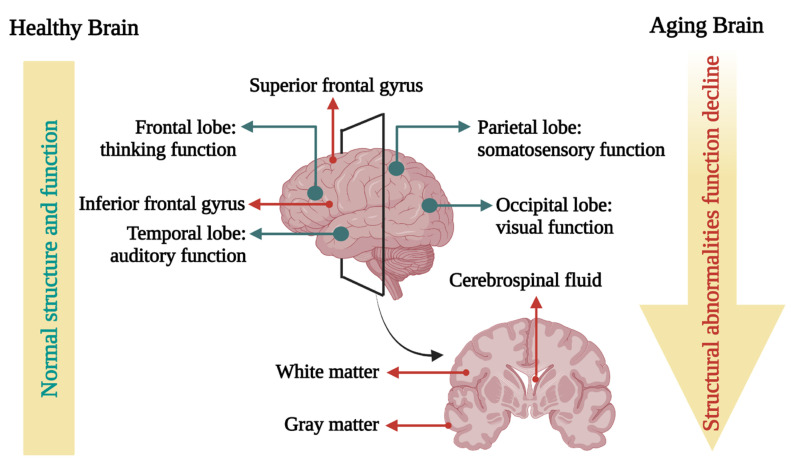
Hallmarks of brain aging.

Aging and pathology are associated with significant changes in the complex microstructure of the brain, which lead to cognitive decline. With age, the brain undergoes significant atrophy, that is, loss of brain tissue volume [10]. These changes directly or indirectly result in functional deficits, including memory loss and reduced motor and behavioral performance. Initially, these brain aging-related changes occur primarily at the cellular level and include reduced metabolic activity and ischemia, which then gradually manifest as changes in brain structure at the tissue and organ levels [11]. Specifically, upon aging, the gray matter density of the human brain decreases rapidly, particularly in the temporal and frontal lobes, and this degenerative change is associated with delayed myelin formation [12]. White matter volume increases slightly in the first half of the life cycle followed by a more pronounced reduction. Anatomical analysis of the cortical surface shows that the width of the cerebral sulcus is positively correlated with age and is accompanied by cognitive decline in the elderly [13,14]. Age-related cortical thickness atrophy is most pronounced in the triangle of the superior frontal gyrus, paracentral gyrus, and inferior frontal gyrus, as revealed by whole-brain cortical thickness testing [15]. Analysis of longitudinal magnetic resonance images shows that the ventricles gradually expand with age [16]. Together, these observations show that the normal aging process triggers a series of structural changes in the brain, including shrinkage, widening of the sulci, enlargement of the ventricles, and a reduction of gray and white matter volumes (Figure 1). Despite the advances in our understanding of brain structure and function, further studies on the cognitive and molecular cellular changes are needed before pharmacological interventions.

## 3. How do Herbal/Natural Compounds Respond to Molecular Changes during Brain Aging?

**Figure 2:**
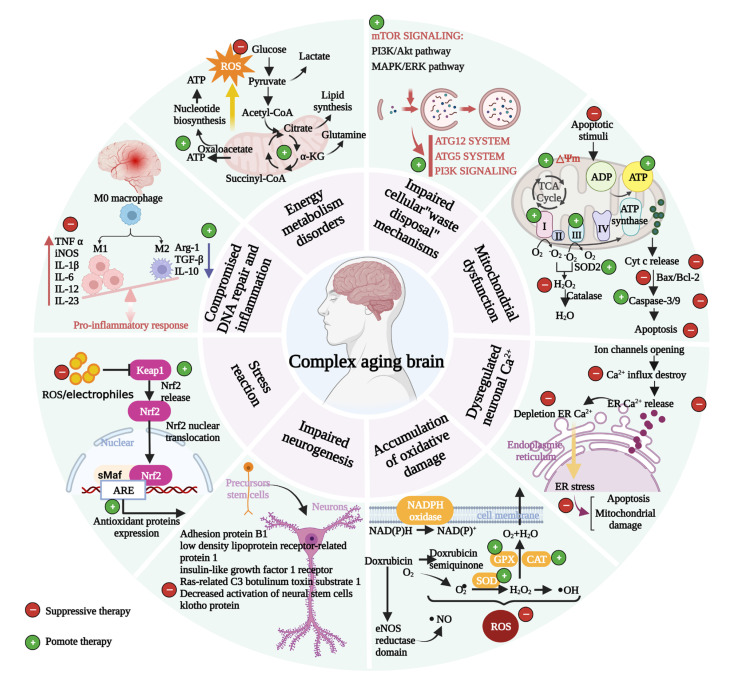
Intervention strategies for herbal/natural compounds based on brain aging characteristics. In the aging state, multiple mechanisms are impaired due to oxidative stress and impaired stress responses, leading to the accumulation of brain waste, mitochondrial dysfunction, dysregulation of energy metabolism, impaired DNA repair, and inflammation. Included is a brief list of targets and alterations produced by herbal or natural compound interventions. Green circles represent promotive effects and red circles represent inhibitory effects. Keap1: kelch-like ECH-associated protein-1; Nrf2: nuclear regulatory factor 2; ARE: antioxidant response element; NADPH: reduced form of nicotinamide-adenine dinucleotide phosphate; eNOS: endothelial nitric oxide synthase; ER: endoplasmic reticulum; Bax: BCL2-Associated X; Bcl-2: B-cell lymphoma-2; ATP: adenosine triphosphate; ADP: adenosine diphosphate; mTOR: mammalian target of rapamycin; ATG5, 12: autophagy-related proteins 5, 12; PI3K: phosphatidylinositol 3-kinase; Akt: protein kinase B; MAPK: mitogen-activated protein kinase; ERK: extracellular signal-regulated kinase; ROS: reactive oxygen species; M0, M1, M2: microglia resting state, pro-inflammatory cells, anti-inflammatory cells; IL-1β1β or 6, 10, 12, 13: interleukins-1β or 6, 10, 12, 13; TNF α: tumor necrosis factor α; Arg-1: Arginase-1; TGF-β: transforming growth factor-β; ΔΨm: mitochondrial membrane potential symbol; I, II, III, and IV: mitochondrial respiratory chain complex, ubiquinone oxidoreductase, succinate dehydrogenase, cytochrome c oxidase, cytochrome c reductase, respectively.

Previous studies of the aging brain at the cellular and molecular levels [17] have revealed many distinct features. Here, we review the main features of brain aging, focusing on the effects of herbal/natural compounds on the following age-related processes and changes: (1) mitochondrial dysfunction, (2) accumulation of oxidative damage, (3) energy metabolism disorders, (4) cell “waste disposal” mechanisms (autophagic lysosome and proteasome function), (5) stress response, (6) compromised DNA repair disorders and inflammation, (7) dysregulated neuronal Ca^2+^ homeostasis, and (8) impaired neurogenesis (Figure 2).

### 3.1. Mitochondrial Dysfunction

To date, several synthetic drugs that improve mitochondrial function have been developed to overcome cognitive impairment. However, there are no natural compounds that modulate synaptic plasticity by directly targeting mitochondria. Mitochondria are a major target of aging and play an important role in the age-dependent degeneration of the human brain; however, studying mitochondria in aging human neurons has been challenging. While current data suggest that mitochondrial dysfunction plays a crucial role in aging and neurodegeneration [18], it is unclear whether it plays a major role in the initial stages of disease or is secondary to other phenomena. Cognitive impairment and mitochondrial dysfunction are key characteristics of aging [19]. A large amount of data (Table 1) show that herbal/natural compounds exert a neuroprotective effect via the following actions: (1) specific modulation of mitochondrial respiratory components to ameliorate mitochondrial dysfunction, thereby alleviating cognitive impairment in aged animals [20]; (2) preventing mitochondria from releasing apoptotic factors (caspase and Bcl-2 family proteins), thereby suppressing the apoptotic cascade and reducing neuronal apoptosis or necrosis caused by aging; (3) improving cognitive function by modulating intracellular energy metabolism through mitochondria, in turn regulating synaptic plasticity-related proteins and structures and promoting the release of neurotrophic factors involved in learning and memory; and (4) enhancing the activity and expression of antioxidants, activating signaling molecules to promote the clearance of mtROS, and ultimately the protection of neuronal/synaptic and cognitive functions to regulate oxidative stress. These findings suggest that herbal/natural compounds as modulators of mitochondrial function may be optimal therapeutic targets for delaying brain aging.

### 3.2. Accumulation of Oxidative Damage

During aging, neurons accumulate dysfunctional proteins and mitochondrial aggregates caused by oxidative imbalance, that is, the increased production of reactive oxygen species (ROS) and/or decreased antioxidant defenses. Resveratrol studies have shown that abnormal nitric oxide-mediated oxidative damage is associated with microvascular dysfunction in the aging cerebral cortex. Notably, resveratrol targets brain microvascular endothelial cells and significantly improves neurovascular coupling [31]. Drugs that decrease peroxynitrite anions can also delay aging and disease progression [32]. Similarly, recent studies show that antioxidants protect brain function in aged rats by lowering the peroxynitrite concentration [33]. Enhancing antioxidant defenses and preventing lipid peroxidation are key to increasing resistance to neuronal protein aggregation. Studies on saponins from *Panax japonicus* (SPJ) show that they can reverse senescence-induced decreases in Fork head box O3 (FOXO 3) and superoxide dismutase 2 (SOD2) and reduce apoptosis in senescent rat cortical and hippocampal cells [34].

### 3.3. Impaired Biological Function of Lysosomes and Proteasomes in Neurons

One of the most salient features of aging is the decline in protein homeostasis [35]. Misfolded, aggregated, and damaged proteins are removed by the proteasome or cleared by the autophagy–lysosome pathway [36]. As a key organelle in cellular degradation, lysosomes exhibit age-related changes [37]. Increasing autophagic activity and maintaining normal autophagic flux in neurodegenerative diseases have emerged as new research hotspots and therapeutic targets. A report on the ginsenoside Rg3 showed that resistance to neurotoxicity was associated with enhanced autophagic flux, autophagic protein levels, and the number of autophagic vesicles in neuronal cells [38]. Another study showed that increasing autophagic flux by promoting interactions between autophagic axon transport-related proteins and inducing lysosomal–autophagosome fusion is essential for protection against neuronal aging [39]. These lines of evidence provide additional support for a critical modulatory role of herbal/natural compounds in lysosomal–autophagic biological functions associated with aging. Amitriptyline is a tricyclic antidepressant that is used to treat major depressive disorder and depressive symptoms associated with various neurological disorders. It has been reported that amitriptyline regulates lysosomal localization by activating the PI3K/Akt/mTOR pathway and beclin-1 acetylation, thereby interfering with the fusion of autophagosomes and lysosomes and increasing the interaction between the proteins Arl8, SKIP, and kinesin [40]. Similarly, epimedoside effectively improves the morphology of cortical and hippocampal neurons in aging rats, likely by enhancing neuronal autophagy levels by activating AMPK and inhibiting the activation of mTOR, thereby inhibiting serine phosphorylation at the ULK1 (Ser757) site [41]. Many of the therapies targeting the lysosomal pathway in neurodegenerative diseases rely on the mTOR signaling pathway [42], and most of these drugs have antidepressive actions (Table 2). However, these drugs generally have severe side effects and are not suitable for long-term use or disease prevention, and, therefore, there is an urgent need for alternative medications. Although herbal/natural compounds overlap with most conventional therapies for neurodegenerative diseases in terms of the molecular pathways targeted, such as the mTOR signaling pathway, traditional medicines have the advantage of low toxicity. The peroxisome is another organelle containing enzymes that degrade peroxides [43]. It has been shown that aging exacerbates the decrease in the number of peroxisomes in neuronal cells in the AD model, and that a certain number of peroxisomes is needed to maintain normal brain functions [44]. However, the role of herbs and natural compounds in brain aging-related diseases is largely unknown, and further study may uncover new therapeutic directions.

### 3.4. Dysregulated Neuronal Ca^2+^ Homeostasis

Ca^2+^ is involved in multiple signaling and organellar homeostatic pathways and is mainly stored in the lysosome and endoplasmic reticulum (ER). During aging, excessive oxidative stress leads to impaired Ca^2+^ handling by the ER, resulting in ER stress [63]. An increasing number of studies show that perturbation of ER calcium homeostasis has a great influence on the mechanism of AD. Indeed, extracts of *Allium* sp. (medicinal and aromatic plants) act on the structure of Aβ_1–42_ to prevent Ca^2+^ imbalance and neurotoxicity [64]. A study on phenols from rhubarb, an important component of Chinese herbal medicines, showed that these compounds exert neuroprotective effects in AD by regulating ER stress [65]. Furthermore, in the central nervous system, Ca^2+^ plays an important role in the regulation of electrical activity and neurotransmitter release [66]. It has been reported that cannabidiol (a pant cannabinoid derived from the cannabis plant) protects against the reduction of dendritic spine density and restores synaptic Ca^2+^/calmodulin-dependent protein kinase II activity in primary hippocampal neurons exposed to Aβ_1–42_ [67]. Aging neurons exhibit an imbalance in Ca^2+^ homeostasis that induces not only endoplasmic reticulum stress, but also affects mitochondrial buffering capacity [68,69]. Ca^2+^ overload and accumulation in mitochondria induces the opening of the mitochondrial permeability transition pore and promotes the release of pro-apoptotic factors [70]. Ginsenoside Rf, an active component in ginseng, has been shown to restore normal mitochondrial buffering capacity by significantly lowering Ca^2+^ concentration, ROS levels, and active calpain I expression, thereby reducing apoptosis [71]. Although the function of lysosome-dependent Ca^2+^ homeostasis in the aging brain remains poorly understood, the ability of lysosomes to regulate cytosolic Ca^2+^ in non-neuronal cells suggests that lysosomes may have a similar role in neurons [66].

### 3.5. Stress Response

Adaptive stress response signaling pathways are impaired during aging, thus making neurons vulnerable to damage and neurodegenerative diseases [72]. A recent study showed that neuroprotective phytochemicals modulate adaptive cellular stress response pathways in neurons by upregulating the expression of neurotrophic factors, antioxidant enzymes, and proteins involved in cellular energy metabolism [73]. In animal models of neurodegenerative disease, various herbal and natural compounds have been shown to protect neurons from dysfunction and degeneration, including folic acid [74], thiamin [75], curcumin [76], epigallocatechin-3-gallate [77], lead flavin [78], and resveratrol [79]. The signaling pathways activated by these compounds include the nuclear regulatory factor 2 (NRF2)–antioxidant response element (ARE) pathway [80], the Ca^2+^–cyclic AMP response element binding protein (CREB) pathway, and the FOXO pathway. Neuroprotective regulatory molecules include neurotrophic factors (e.g., BDNF), protein chaperones (e.g., heat shock proteins [81] and glucose regulatory proteins [82]), antioxidant enzymes (e.g., Mn-SOD, heme oxygenase 1, and NADH quinone oxidoreductase 1), energy regulatory proteins (e.g., mitochondrial uncoupling proteins), and DNA repair proteins (e.g., AP endonuclease 1). The discovery of these herbal and natural compounds should spur the development of drugs that target stress response pathways with high specificity and low toxicity.

### 3.6. Compromised DNA Repair Disorders and Inflammation

The gradual accumulation of DNA damage is associated with cellular and organismal aging, usually manifested by DNA loss and decreased DNA repair capacity. Brain mitochondrial DNA deletion, reduced mitochondrial respiratory chain complex I and IV activity, and decreased ATP synthesis in aged rats can be ameliorated by the Chinese herbs lycium and semen cuscutae [83]. A study found that phosphorylated H2AX (γ-H2AX, Histone H2AX phosphorylation) is a powerful new marker for the detection of DNA double-strand breaks [84]. Elevated γ-H2AX levels in the brains of senescent mice can be significantly reduced by resveratrol, preventing the accumulation of DNA damage [85]. Furthermore, the decrease in DNA repair capacity during aging is associated with increased damage to mitochondrial DNA, and the key pharmacological targets are the DNA repair enzymes DNA polymerase γ and 8-hydroxyguanine glycosidase 1 (OGG1) [86]. In addition, base excision repair in neurons is equally important for repairing oxidative DNA damage. The natural antioxidant quercetin prevents tert-butyl hydroperoxide (t-BHP)-induced DNA strand breaks and cell damage, and in vitro studies show that this effect is mediated by an increase in DNA repair enzyme activity, but no change in the expression of the OGG1 was detected [87].

Neuroinflammation is characterized by the activation of neuroglia, including astrocytes and microglia, which are the primary immune cells in the brain. Once activated, microglia are involved in the inflammatory response, promoting the release of cytokines and chemokines, nuclear factor κB (NF-κB), tumor necrosis factor α (TNF-α), adhesion molecules, and other factors [88]. Resveratrol inhibits microglia activation by promoting microglia polarization to the M2 phenotype through PGC-1α(peroxisome proliferator activated receptor γ coactivator 1α). PGC-1α not only inhibits microglial M1 marker expression by inhibiting NF-κB activity, but also increases M2 marker expression by co-activating the transducer and activator of transcription 6 (STAT6) and STAT3 pathways [89]. Notably, neuroinflammation is regulated by microglia, which can have both neurotoxic and neuroprotective effects. For example, curcumin can improve the cognitive behavior of lipopolysaccharide-induced neuroinflammation by inhibiting the activation of M1-type microglia [90]. Flavonoids in plants also have the ability to modulate microglial activation (inhibit M1-type activation) and reduce various inflammatory factors in the brain [91]. There is accumulating evidence that activation of inflammatory processes is a hallmark of all neurodegenerative diseases. Elevated levels of pro-inflammatory eicosanoids, cytokines, and chemokines, as well as activated microglia and astrocytes, have been found in the AD brain, even in the very early stages of the disease [92,93,94,95]. Treatment with the herbal medicine icariin protects against damage to dopaminergic neurons by inhibiting NF-κB pathway activation and suppressing microglial activation and pro-inflammatory factor production [96,97].

### 3.7. Impaired Neurogenesis

Neurogenesis is a complex process that involves the proliferation of neural stem cells and their differentiation to progenitor cells that migrate to functional areas, undergo continuous plastic changes, and establish synaptic connections with other neurons to produce neurological functions. However, the degree of neurogenic damage increases with age [98]. The neuroprotective and neurogenic properties of the non-saponin component (NFP) rich in Korean red ginseng polysaccharides are observed in both aged and AD brains, especially on neuronal death and adult hippocampal neurogenesis. Low-density lipoprotein receptor-related protein 1, insulin-like growth factor 1 receptor, and Ras-related C3 botulinum toxin substrate 1, which are affected by NFP treatment, play important roles in neuronal cell growth and neurodevelopment [99]. Oxidative stress, impaired DNA repair, and inflammation may also contribute to age-related reductions in neurogenesis [100]. *Gastrodiae Rhizoma* (Tianma) was shown to inhibit oxidative stress and increase hippocampal neurogenesis in both D-gal-exposed cells and brain tissue from senescent mice [101]. Although aging neural progenitor cells exhibit reduced mitochondrial oxidative metabolism and impairment of mitochondrial function, which can lead to neurogenic damage, the effects of herbal or natural products have not yet been studied [102]. Recent evidence suggests that neurogenesis can slow the aging process [103]. An important goal for future studies is to identify herbal or natural compounds that slow brain aging.

### 3.8. Energy Metabolism Disorders

Impaired glucose and lipid metabolism in peripheral tissues and brain cells frequently occurs during aging, as evidenced by an impaired ability of intracellular insulin to increase glucose transport and elevated circulating glucose concentrations [104]. Aging human neuronal cell lines also exhibit reduced glucose utilization, and rosemary extract stimulates glucose uptake in neuronal cell lines, an effect that is independent of insulin signaling [105]. Furthermore, rodents with mild cognitive impairment and AD exhibit impaired glucose metabolism resulting from oxidative stress-mediated dysfunction of neuronal glucose transport proteins [106]. In an in vivo study, the herbal mixture Shenzhiling oral liquid ameliorated hippocampal neuronal degeneration, reduced Aβ peptide aggregation, and improved cellular ultrastructure by modulating insulin signaling pathways and increasing glucose uptake, upregulating glucose transport proteins (GLUT1 and GLUT3), and enhancing glycolysis [107]. The metabolic health of the gut can be relayed to the brain via neural and circulatory pathways. Although the mechanisms of this interaction remain unclear, numerous neurological disorders are associated with enteric malnutrition [108]. Therefore, targeting the gut–brain axis may be an effective strategy for controlling glucose metabolism [109], but reports on herbs or natural compounds are lacking.

Neuronal insulin resistance is a major risk factor for age-related metabolic diseases such as diabetes and cardiovascular disease. Although reports suggest that insulin resistance can be ameliorated by downregulating phosphatase and tensin homolog (PTEN) [110], other studies suggest that modulating energy metabolism and synaptic function is preferred to reduce the risk of cognitive decline associated with metabolic diseases such as diabetes [111]. In addition to the glucose metabolic dysfunction, the metabolism of several lipid species is also altered during aging [112]. For example, the herbal ginsenoside mixture and Tripterygium glycosides can increase nematode lifespan by enhancing lipid metabolism and reducing fat accumulation [113]. Metabolic factors can accelerate or slow brain aging, and interventions that support neuronal bioenergetics have considerable potential for slowing brain aging and preventing neurodegenerative diseases.

## 4. Herbal/Natural Compounds for the Treatment and Prevention of Neurological Disorders

### 4.1. AD

Mitochondrial dysfunction and increased oxidative stress are key features of the AD brain. These pathological characteristics can be rescued by traditional Chinese medicines in the AD mouse model, manifested as increased basal respiration in hippocampal mitochondria. Moreover, the transcription factor Nrf2 can activate protective genes to regulate the endogenous antioxidant response pathway [114,115]. The characteristic pathological changes of AD are extracellular senile plaques composed of beta amyloid deposits and intracellular neurofibrillary tangles formed of hyperphosphorylated tau protein, as well as neuronal loss with glial cell proliferation. A growing number of studies on these pathological alterations suggest that protection of neurovascular coupling is key, specifically by modulating neurovascular RAGE and ERK/CREB/BDNF pathways to reduce neurovascular oxidation and regulate microvascular function with the aim of maintaining neurovascular unit integrity and enhancing the cholinergic system [116].

**Figure 3:**
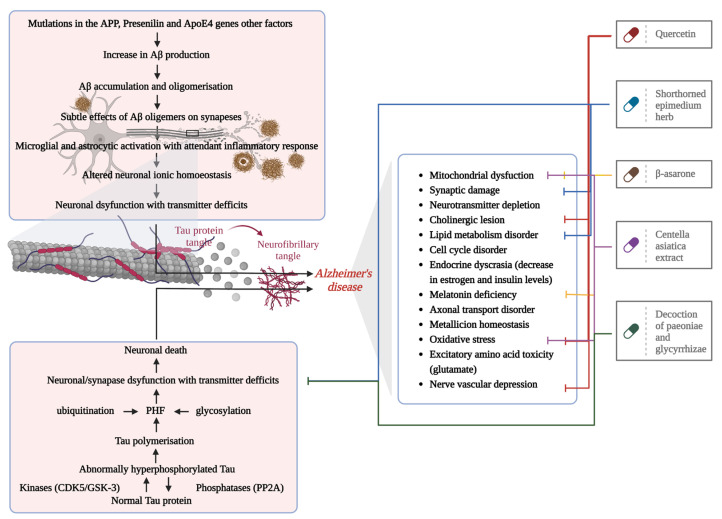
Pathological features of AD and herbal/natural compound strategies. The hallmark pathological features of AD are Aβ plaques and tau-containing neurofibrillary tangles. Aβ plaques and tau aggregation can lead to oxidative stress, mitochondrial dysfunction, and the induction of neuronal markers of aging. Herbs and natural compounds target the markers of aging to slow the disease process. ApoE4: apolipoprotein E4; APP: amyloid precursor protein; PHF: paired helical filament; CDK5: cyclin-dependent kinases-5; GSK-3: glycogen synthase kinase-3.

In AD, reducing neuritic atrophy and synaptic loss is considered the best strategy to improve memory impairment [117,118]. A number of studies suggest that maintaining neuronal functional homeostasis and brain energy metabolism have long-term benefits for the AD brain [119,120]. Icariin is the main active ingredient of Epimedium, which has attracted increasing attention because of its unique pharmacological effects in degenerative diseases. In in vitro and in vivo AD models, the enhanced mitophagic activity induced by icariin combined with β-asarone promotes the clearance of Aβ and tau [121]. Although the mechanisms by which neurons are able to resist the neurotoxic effects of Aβ in older adults remain unclear, experimental data suggest a role of neurotrophic factor signaling and adaptive cellular stress response pathways [122]. Another drug with significant neuroprotective effects, resveratrol, regulates glycogen synthase kinase-3 (GSK3) and enhances the transport of thyroxine proteins to clear tau and maintain normal synaptic function [123]. Although several therapeutic strategies for AD have been evaluated (Figure 3), the use of effective supplements or drugs remains limited.

### 4.2. PD

**Figure 4:**
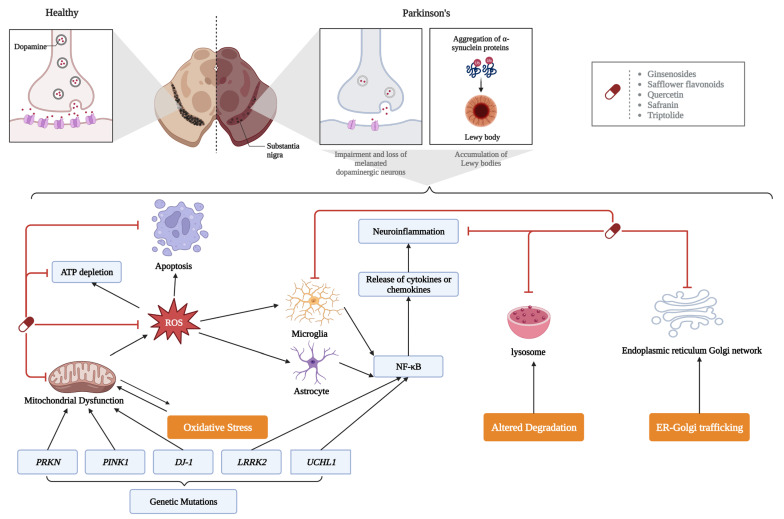
Pathological features of PD and herbal/natural compound strategies. In PD, the aging process leads to the accumulation of intracellular neurotoxic α-synuclein. In turn, the accumulation of α-synuclein exacerbates the aging process. Gene mutations, oxidative stress, endoplasmic reticulum stress, lysosomal degradation imbalance, neuronal dysfunction, and apoptosis play major roles in disease pathogenesis and progression. ATP: adenosine triphosphate; PRKN: parkin RBR E3 ubiquitin protein ligase; PINK1: PTEN-induced putative kinase 1; DJ-1: Parkinson disease protein 7 (PARK7); LRRK2: leucine-rich repeat kinase 2; UCHL1: ubiquitin C-terminal hydrolase L1; NF-κB: nuclear factor κB.

In PD, oxidative damage, dysregulation of neural Ca^2+^ homeostasis, impaired DNA repair, impaired adaptive cellular stress response, and neuroinflammation all occur in brain regions affected by the disease, and these age-related alterations increase neuronal vulnerability, α-synuclein pathology, and mitochondrial and autophagic dysfunction [124]. Currently, pharmacotherapy is the predominant treatment for PD. Dopamine receptor agonists and the dopamine precursor levodopa alleviate PD symptoms. However, with long-term use, these drugs can cause severe complications, such as dyskinesia. Therefore, the focus of current research has shifted from dopaminergic to non-dopaminergic strategies to reduce dopaminergic complications [125]. Herbal/natural compound therapies can overcome the limitations of current dopaminergic therapies. For example, safranin [126], syringic acid [127], gallic acid [128], ellagic acid [129], and triptolide [130] have been used in PD treatment or prevention studies. Herbal/natural compounds have been shown to have multifaceted actions in PD treatment and prevention (Figure 4). α-synuclein overexpression or aggregation is a trigger for PD pathogenesis, and saffron flavonoid extract (SAFE) reduces α-synuclein abnormalities and reactive astrocyte proliferation. In addition, the inhibitory effect of SAFE on nigrostriatal diffusion parameters provides clues to neuronal loss and astrocyte activation [131]. Apoptosis occurs in dopaminergic neurons of PD patients and nigrostriatal cells of PD animal models, and some herbal/natural compounds have been shown to be effective in reducing apoptosis in PD cells and animal models [132], providing a basis for clinical translation. An increasing number of herbal/natural compounds have recently been shown to have promising effects in PD treatment or prevention by ameliorating mitochondrial dysfunction, including quercetin [133] and ginseng [134]. Although levodopa is the most effective anti-PD agent, with better efficacy for hypokinesia and hypertonicity, it is less effective for tremor, especially in 15% of cases where levodopa is ineffective. Therefore, the application of herbal/natural compounds and cell transplantation, especially embryonic stem cells, may offer new hope for the treatment of PD [135].

## 5. Materials and Methods

This study was conducted using the PubMed database for literature between 2000 and 2023. The keywords used were “aging “, “brain”, “aged-related diseases”, “herbal”, “natural compounds”, “neuronal”, “oxidative damage”, “autophagy”, “energy metabolism”, “stress response”, “DNA repair”, “neuronal Ca^2+^”, “neurogenesis”, and “inflammation”. We initially screened for review articles, and the mechanisms of herbal and natural compounds were mainly researched after screening for original research articles. Screening was performed according to the above criteria and 139 articles were finally included in this review.

## 6. Conclusions and Outlook

Research in the fields of aging and neuroscience has revealed multiple features of brain aging at the molecular, cellular, and systemic levels. Due to the complexity of the aging process, the development of drugs that promote optimal brain function and resistance to disease is a formidable undertaking. The lack of effective and low-toxicity drugs for age-related brain diseases is a challenge that must be tackled in earnest. Herbal medicines and natural compounds might be a better option for treating brain diseases [136]. Although herbal/natural compounds have multi-target and multi-systemic therapeutic actions, these can be divided into organ, tissue, and cellular-level effects, in addition to disease-specific targets. An appropriate diet is essential for brain health. One study found that the Mediterranean diet reduces the risk of stroke in women and improves cognitive performance in older patients [137]. In addition, it is effective in preventing the development of AD-related biomarkers. However, these diets need to be based on a doctor’s prescription as excessive or poor diets are more likely to cause brain damage. A growing amount of data suggests that regular intermittent metabolic switching can counteract the features of aging [138]. Therefore, brain health can be enhanced by a combined model of medication and intermittent regular dieting, thereby allowing the brain and other systems to perform optimally.

Nicotinamide mononucleotide (NMN), a natural compound present in the body and continuously metabolized, is the most promising anti-aging drug. The naturally declining nicotinamide adenine dinucleotide (NAD+) levels with age can be replenished by NMN supplementation [139]. Similarly, levodopa is a precursor drug for dopamine, which is converted to dopaminergic by the action of dopamine decarboxylase and acts as a dopaminergic supplement [140]. These previous studies led us to question whether direct supplementation of a single disease-specific target or component is the best therapeutic option. Due to the previous experience with L-dopa [141], we posited that an insufficient or excessive dose could inflict a greater burden or even toxic side effects on the organism. In addition, some investigations have shown that the disease is subject to lagging in vivo indicators [142], that is, symptoms precede indicators of disease. Therefore, optimizing the timing of drug intervention according to the different pathological characteristics and stages of the disease is also important.

Another major challenge in the development of drugs to combat brain aging is the difficulty of drugs in crossing the blood–brain barrier (BBB) to exert their therapeutic effects on neurons. For example, some flavonoids and their metabolites must traverse the BBB and enter the brain parenchyma to acquire neuroavailability. Fortunately, some flavonoids are retained longer in the neural tissue than in plasma, which greatly increases their action in the brain [143,144]. However, the BBB is the major barrier for delivery of some natural compounds (e.g., curcumin) [145], requiring synthetic nanomedicines for delivery. Potential clinical applications for nanocurcumin are emerging and could overcome the therapeutic limitation of free curcumin, and thus ameliorate cellular and organ dysfunctions associated with aging [146].

In this review, we discussed the anti-aging potential of herbs and natural compounds with a focus on the aging brain, with the aim of affording insight into strategies for the prevention and treatment of brain diseases. We hope that this will provide a basis for the development of herbal and natural compounds for treating aging-related disorders of the brain.

## Figures and Tables

**Table 1:** Treatment of the aging brain targeting the mitochondrial pathway.

Drugs	Mechanism	Outcomes	Reference(s)
*Agaricus blazei* extract	Regulate mitochondrial respiration	Increased mitochondrial respiratory enzyme activity (NADH: ubiquinone oxidoreductase, succinate dehydrogenase, cytochrome c oxidase)	[21]
Ginkgo biloba extract			[22]
Ganoderma lucidum			[23]
Curcuminoids			[24]
Schisandra Extract			[25]
Diosgenin	Prevent the release of apoptotic factors	Inhibited senescence-induced mitochondria-dependent apoptotic pathway (Bax, cytochrome c, active caspase-9 and active caspase-3) and IGF-1-PI3K-AKT survival pathway	[26]
Baicalin			[27]
Baicalein			[27]
Cyanidin 3- O-β-Galactoside	Energy metabolism	Increased the levels of N-acetyl-l-leucine, N-acetyl-l-tyrosine, and methionine sulfoxide; reduced the levels of both hyodeoxycholic acid and chenodeoxycholic acid	[28]
Astaxanthin			[29]
Honokiol	Mitochondrial oxidative stress	Increased activity and expression of SOD2 and activated Sirt3 to promote mtROS clearance	[30]

NADH: Nicotinamide adenine dinucleotide; IGF-1-PI3K-AKT: insulin-like growth factor 1-Phosphatidylinositol 3-kinase-Protein kinase B; SOD2: superoxide dismutase 2; Sirt3: sirtuin3; mtROS: mitochondrial reactive oxygen species.

**Table 2:** Treatment of neurodegenerative diseases targeting the lysosomal pathway.

Disease	Drugs	Mechanism	Outcomes	Reference(s)
PD	Rapamycin	mTOR-related	Inhibited mTORC1 and S6K; mediated degradation of SNCA/α-synuclein aggregates by GSK3	[45,46,47]
	Temsirolimus			
	6-Bio			
	Trehalose	mTOR-unrelated	Maintained glucose transporter-1 and promoted protein stability; inhibited Ca^2+^ overload	[48,49,50]
	Minoxidil			
	Verapamil			
	Acidic nanoparticles	Lysosomal	Restored lysosomal acidification; increased glucosylcerebrosidase; inhibited glucosylceramide synthase	[51,52,53,54,55]
	Ambroxol			
	Isofagomine			
	Venglustat			
AD	Carbamazepine	mTOR-related	Inhibition of mTOR activity	[56,57,58]
	Latrepirdine			
	Temsirolimus			
	Lithium	mTOR-unrelated	Involved in IP3; as an activator of SIRT1; antagonized NMDAR	[59,60,61]
	Resveratrol			
	Memantine			
	Metformin	Lysosomal	Induced CMA, activation of TAK1-IKK α/β signaling	[62]

PD: Parkinson’s disease; AD: Alzheimer’s disease; 6-Bio: A autophagy modulator; GSK3: Glycogen synthase kinase-3; SNCA/α-synuclein: Synucleopathies-related proteins; mTOR: mammalian target of rapamycin; S6K: Ribosome S6 protein kinase; IP3: myo-inositol-1,4,5-trisphosphate; NMDAR: N-Methyl-d-aspartate receptor; CMA: chaperone-mediated autophagy; TAK1-IKK α/β: I-kappa-B kinaseα/β/Transforming growth factor-beta-activated kinase1.

## Data Availability

Not applicable.

## Abbreviations

AD	Alzheimer’s Disease
ATP	Adenosine triphosphate
Arg-1	Arginase-1
ARE	Antioxidant response element
ADP	Adenosine diphosphate
ATG 5	Autophagy-related proteins 5
ATG 12	Autophagy-related proteins 12
AKT	Protein kinase B
Arl8	A small ADP-ribose-factor-like RAS family GTPase that mediates lysosomal transport driven by kinesin
AMPK	AMP-activated protein kinase
APP	Amyloid precursor protein
ApoE4	ApolipoproteinE 4
Bax	BCL2-Associated X
Bcl-2	B-cell lymphoma-2
BDNF	Brain-derived neurotrophic factor
BBB	Blood–brain barrier
CAT	Catalase
CREB	Cyclic AMP response element binding
CDK5	Cyclin-dependent kinases-5
DJ-1	Parkinson disease protein 7, PARK7
DA	Dopamine
ERK	Extracellular signal-regulated kinase
ERS	Endoplasmic reticulum stress
ER	Endoplasmic reticulum
FOXO	Fork head box class O
GPX	Glutathioneperoxidases
GSK-3	Glycogen synthase kinase-3
iNOS	Inducible nitric oxide synthase
IL-1β	Interleukins-1β
IL-6	Interleukins-6
IL-10	Interleukins-10
IL-12	Interleukins-12
IL-13	Interleukins-13
Keap1	Kelch-like ECH-associated protein-1
LRRK2	Leucine-rich repeat kinase 2
MAPK	Mitogen-activated protein kinase
mTOR	Mammalian target of rapamycin
Nrf2	Nuclear regulatory factor 2
NADPH	Reduced form of nicotinamide-adenine dinucleotide phosphate
NF-κB	Nuclear factor κB
NFP	Non-saponin component
NMN	Nicotinamide mononucleotide
NAD+	Nicotinamide adenine dinucleotide
OGG1	8-hydroxyguanine glycosidase 1
PD	Parkinson’s disease
PI3K	Phosphatidylinositol 3-kinase
PGC-1α	Peroxisome proliferator-activated receptor-γ coactivator-1α
PHF	Paired helical filament
PRKN	Parkin RBR E3 ubiquitin protein ligase
PINK1	PTEN induced putative kinase 1
ROS	Reactive oxygen species
SOD	Superoxide dismutase
SPJ	Saponins of *Panax japonicus*
SKIP	Skeletal muscle and kidney-enriched inositol polyphosphate 5-phosphatase
STAT 3	Signal transducer and activator of transcription 3
STAT 6	Signal transducer and activator of transcription 6
SAFE	Saffron flavonoid extract
STAT6	Signal transducer and activator of transcription 6
TNF-α	Tumor necrosis factor α
TGF-β	Transforming growth factor-β
TCA	Tricarboxylic acid cycle
UCHL1	Ubiquitin C-terminal hydrolase L1
α-Syn	alpha-synuclein
α-KG	alpha-ketoglutarate
γ-H2AX	Histone H2AX phosphorylation
